# Copper-Modified Cellulose Paper: A Comparative Study of How Antimicrobial Activity Is Affected by Particle Size and Testing Standards

**DOI:** 10.3390/ijms26020480

**Published:** 2025-01-08

**Authors:** Sara Ramírez, Fabian Zúñiga, Alejandra Amenábar, Paulina Contreras, Viviana Benavides, Javiera Norambuena, Jessica Martínez, Nataly Silva

**Affiliations:** 1 Facultad de Diseño, Universidad del Desarrollo, Avenida Plaza 680, Las Condes 7610658, Santiago, Chileaamenabar@udd.cl (A.A.); paulinacontreras@udd.cl (P.C.);; 2 Centro de Medicina Regenerativa, Facultad de Medicina, Clínica Alemana-Universidad del Desarrollo, Avenida Plaza 680, Las Condes 7610658, Santiago, Chile

**Keywords:** copper, cellulose, paper, substrate, microparticles, nanoparticles, antibacterial, antimicrobial

## Abstract

This study aims to provide evidence that when testing cellulose paper modified with copper particles (CuPs), the particle size and the analysis method influence the antimicrobial activity observed by this material. Commercial CuPs of nanometric size (2.7 nm, CuNPs) and micrometric size (2.5 µm, CuMPs) were used to modify cellulose paper sheets. CuPs were incorporated during the pulp disintegration phase (stage 1) of the sheet formation process, according to the ISO 5269-1:2005 standard. Modified paper sheets retained 16% and 14% of CuNPs and CuMPs, respectively. Additionally, CuPs were distributed randomly on the fiber surfaces, often forming aggregates. Finally, the antimicrobial activity of the modified paper sheets was evaluated using ISO 20645:2004 and ISO 20743:2013. The results showed that the antimicrobial activity assessed using each standard method is conditioned by the mechanism of action of the CuPs and, therefore, by their size. It was concluded that ISO 20645:2004 is suitable for evaluating the antibacterial effect of paper/CuNPs, as nanoparticles diffuse from the paper and are released into the culture medium. In contrast, ISO 20743:2013 can be used for both CuNP- and CuMP-based paper, as it evaluates the antibacterial effect based on the direct interaction between the copper particle and the bacteria.

## 1. Introduction

The growing threat of antimicrobial resistance has driven the search for alternative materials with inherent antimicrobial properties, thus offering promising solutions to mitigate microbial contamination and the spread of infectious diseases [1]. Among these materials, copper-based compounds, particularly copper nanoparticles (CuNPs) and copper microparticles (CuMPs), have demonstrated remarkable antimicrobial properties owing to their unique physicochemical properties and multifaceted mechanisms of action [2]. These properties make copper an attractive option for incorporation into various substrates, including paper [3,4], fabric [5], and polymers [6].

Among these substrates, paper is particularly noteworthy due to its widespread use and the inherent difficulty in sanitizing it effectively, as the latter makes it a potential vector for fomite-based pathogen transmission [7,8,9,10]. This issue is of considerable importance given the global consumption of paper, which reached approximately 420 million tons in 2023 and is projected to increase to 476 million tons by 2032 [11,12]. The paper manufacturing sector also plays a critical economic role, contributing significantly to global Gross Domestic Product (GDP) and employing millions of individuals worldwide [13,14]. In Europe, this industry directly employs approximately 180,000 individuals, generating a turnover of EUR 115 billion and adding EUR 25 billion to the EU’s GDP [13]. Globally, the pulp and paper market were valued at USD 357.21 billion in 2023, with projections of USD 391.39 billion in growth by 2032 [14]. These numbers underscore the need for innovative approaches, such as antimicrobial modifications, to enhance the functional properties of paper while addressing its associated risks.

Cellulose paper modified with copper particles (CuPs) has emerged as a promising solution due to its versatility and biocidal activity [4]. Its potential has been explored in a wide array of applications, including photocatalyst [15], bactericide membranes in medicine [16], high-performance textiles [17], conducting paper for electronics [18], antibacterial and anticorrosion films [19], antimicrobial membranes for water treatment [20], and food packaging [21], among others. However, the antimicrobial efficacy of these materials is not determined solely by their chemical composition but is significantly influenced by the size of the incorporated copper particles, which directly affects their mechanism of action and overall performance, as discussed later.

When evaluating antimicrobial properties of such modified materials, reliable and standardized testing is needed. Two widely recognized standards (i) ISO 20645:2004 [22] (ii) ISO 20743:2013 [23]. ISO 20645:2004 relies on the diffusion of antimicrobial agents through an agar matrix, whereas ISO 20743:2013 evaluates the effect of direct contact on antimicrobial activity. However, these methods still have certain limitations associated with their sensitivity depending on the type of modified material and particle sizes of particles used to modify the materials. A study carried out by Contreras et al. reported that there are some challenges in accurately assessing the relationship between particle size and antimicrobial activity, hindering the optimization of these materials for practical applications. Their results revealed that ISO 20743 was more sensitive than ISO 20645 for determining the antimicrobial effects of different papers. Using ISO 20743:2013, it was shown that when direct contact between the microorganism and the sample occurred in suspension, the release and diffusion of copper ions from the paper into the aqueous phase was enhanced. In this case, Cu ions interacted directly with the pathogen’s outer membrane, inducing microbial death upon contact without the need to diffuse through the agar matrix, as observed in disc diffusion assays (ISO 20645:2004). These results highlighted that the release and diffusion of copper ions are influenced by the surrounding medium [24].

This study investigates the applicability of these standards for assessing the antimicrobial performance of paper sheets functionalized with copper particles. The results reveal that the effectiveness of each standard depends on the particle size and mechanism of action of the copper-based material. While ISO 20645:2004 is limited to evaluating CuNPs due to the requirement for diffusion, ISO 20743:2013 proves suitable for assessing both CuNPs and CuMPs, as their antimicrobial action is primarily contact-based. These findings provide critical insights into the appropriate selection of testing methodologies for copper-functionalized materials.

## 2. Results

### 2.1. Characterization

**Figure 1:**
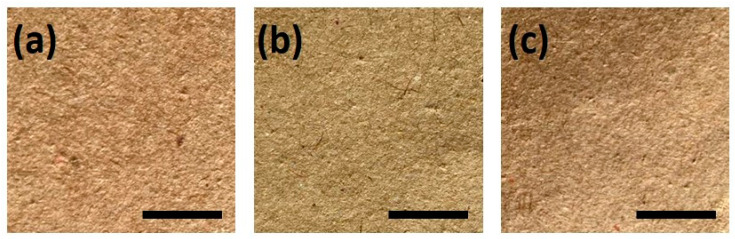
Photograph of the papers produced using the sheet former: (**a**) control paper (unmodified), (**b**) paper/CuNPs, and (**c**) paper/CuMPs. The scale bar corresponds to 1 cm.

Incorporating CuPs during stage 1 of pulp disintegration does not interfere with the paper sheet formation process. Figure 1 shows samples of the paper sheets generated with CuPs alongside the control paper (unmodified paper). It can be observed that the samples modified with CuNPs (paper/CuNPs) exhibit apparent color variations compared to the control. This is confirmed by the CIELAB parameter results shown in Table 1, indicating a decrease in luminosity (L) and a greener hue, likely associated with the partial dissolution of the fine CuNP powder in the aqueous suspension, leading to the release of copper ions. These ions can interact with the cellulose substrate, potentially forming coordination complexes that can alter the material’s properties [25], in this case reducing luminosity. In contrast, copper microparticles (CuMPs), owing to their larger size and lower surface area, exhibit limited dissolution and interaction with both water and cellulose substrate, resulting in minimal changes to the material’s optical properties. It should be noted that the presence of CuMPs slightly reduces the wettability of our cellulose paper. When 20 µL of water was deposited on the surface, all papers began to absorb it instantly, but there were slight differences in the absorption rate. From fastest to slowest, the absorption rates were as follows: paper/CuNPs > paper > paper/CuMPs (Figure S1, Supplementary Materials).

The behavior of our papers aligns with the high moisture sensitivity of cellulose, indicating that the presence of modifying agents can significantly affect this property, as well as other properties such as mechanical and/or adhesion characteristics [26]. These changes have been described in detail previously [4]. Importantly, the presence of CuMPs has negligible effects on the quality of the paper, maintaining the standards of the paper industry.

The amount of copper in the modified paper sheets was determined using atomic absorption spectroscopy, yielding a total of 0.08 g and 0.09 g for CuNPs and CuMPs, respectively. This corresponds to 14% and 16% copper retention relative to the 0.6 g incorporated during stage 1 of pulp disintegration.

**Figure 2:**
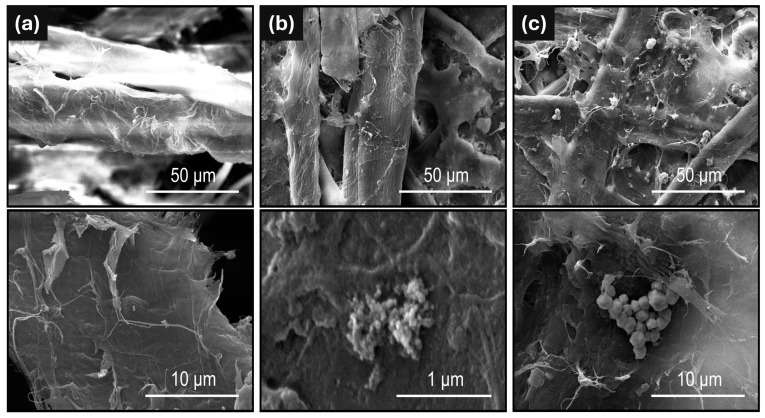
SEM micrographs of (**a**) unmodified paper (control), (**b**) paper/CuNPs, and (**c**) paper/CuMPs.

The paper samples were also analyzed using a scanning electron microscope (SEM), as shown in Figure 2. In Figure 2a, unmodified cellulose fibers are observed as a control. In Figure 2b, cellulose fibers with similar characteristics and areas of color contrast are visible. Only clusters of CuNPs can be seen upon magnification. Due to the equipment’s sensitivity, it is impossible to observe isolated CuNPs modifying the fibers. In Figure 2c, cellulose fibers containing CuMPs are visible and distributed across the surface.

To complete the physicochemical studies, the stability of the interaction between copper particles and cellulose fibers was evaluated. The copper content in the modified papers was initially measured using XRF. The samples were then subjected to an extreme washing process, which involved immersion in water for 10 min, followed by vacuum filtration and washing with three 5 mL aliquots of water [27]. Finally, the copper content was measured again using XRF, and the papers modified with CuNPs experienced a copper loss of 3.52 ± 0.19%, compared to 0.61 ± 0.20% for paper/CuMPs.

### 2.2. Antimicrobial Activity

The results associated with ISO 20645:2004 are shown in Table 2. Only the CuNP-modified paper samples presented an antimicrobial effect against *Staphylococcus aureus* and *Escherichia coli*, generating an inhibition zone of 1.97 ± 0.29 and 4.13 ± 0.29 mm, respectively. The CuMP-modified paper sample did not inhibit the dense growth of bacteria, offering an insufficient antibacterial effect; the same was observed for the control paper.

The results from the antimicrobial activity analysis performed according to ISO 20743:2013 at 0 and 24 h are shown in Table 3. Differing from the results obtained using the ISO 20645:2004 standard, both paper samples modified with CuNPs and CuMPs exhibited strong antimicrobial activity against *S. aureus* and *E. coli* after 24 h of exposure, compared to the observed growth in untreated control paper. The CuNP-modified paper was more effective against both strains than the CuMP-modified paper.

## 3. Discussion

The antibacterial activity of CuPs is primarily attributed to the release of copper ions; therefore, their toxicity is correlated with their concentration and the rate of ion release. When incorporated into a solid matrix such as paper, CuNPs and CuMPs show notable differences in ion release rates, largely due to differences in size and, accordingly, surface area/volume ratios. CuNPs, with a higher surface area to volume ratio, release ions more readily than CuMPs. This increased ion release in smaller nanoparticles often results in higher toxicity relative to larger particles [28]. The diffusion of nanoparticles is closely related to their size; studies focusing on CdSe and silica nanoparticles in gelatin showed reduced diffusion with a particle size increase. In nanoparticles with a diameter of 3.8 nm, the diffusion was in the order of 10–11 m^2^/s, effectively hindering their penetration through the culture media [29].

These results show that both CuNPs and CuMPs demonstrate strong antibacterial effects against both bacterial strains when tested under the ISO 20743:2013 standard, with CuNPs showing potent antimicrobial activity under both standards. Previous studies have shown that CuNPs, due to their small size and large surface-to-volume ratio, tend to be more effective in penetrating bacterial membranes, especially in Gram-positive bacteria, which are more susceptible to copper ions due to their thicker peptidoglycan layer [28].

Our findings reveal that paper modified with CuNPs releases a significantly higher amount of copper (3.52 ± 0.19%) compared to paper modified with CuMPs (0.61 ± 0.20%). This higher release aligns with the strong antimicrobial effect observed for paper/CuNPs when evaluated using the ISO 20645:2004 standard, as this method benefits from the nanoparticles’ ability to diffuse into the culture medium.

In contrast, the ISO 20743:2013 adsorption method was found to be more suitable for assessing the antimicrobial activity of paper/CuMPs. This is due to the larger size of the microparticles (2500 ± 800 nm) and their restricted mobility, as they remain embedded within the paper matrix, resulting in minimal diffusion. Under this method, bacterial suspension is directly applied to the paper’s surface, making the antimicrobial effect primarily reliant on direct contact between the copper particles and the microorganisms, particularly in humid conditions.

These findings highlight the importance of both the particle size and the evaluation method for determining the antimicrobial efficacy of copper-modified cellulose paper, offering valuable insights for optimizing antimicrobial surface designs in various applications.

In the future, it would be important to explore strategies to optimize the uniform distribution of copper particles within the paper matrix, as the observed aggregates may affect the effectiveness of the antimicrobial effect. Similarly, efforts could focus on improving the retention of copper particles in the paper and conducting studies on the durability of antimicrobial properties during prolonged use or reuse, considering factors such as exposure to humidity, high temperatures, and copper particle concentration. Additionally, it would be interesting to evaluate these properties against a broader range of microorganisms and substrates, thereby expanding the potential industrial applications of copper-modified cellulose paper.

## 4. Materials and Methods

### 4.1. Chemicals, Materials, and Bacterial Strains

Spherical copper particles (CuPs) with 99.9% purity, supplied by AINTECH SPA (Santiago, Chile), were used as received. The particles had two distinct diameters, 2.7 ± 0.54 nm and 2500 ± 800 nm, corresponding to copper nanoparticles (CuNPs) and copper microparticles (CuMPs), respectively (Figure S2, Supplementary Materials). *Staphylococcus aureus* ATCC 25923 and *Escherichia coli* ATCC 25922 were grown on using Luria-Bertani Broth agar (Difco, Franklin Lakes, NJ, USA).

### 4.2. Paper Production and Incorporation of Copper Particles

The experimental method used to develop antimicrobial paper preformed as previously reported [3]. Standard paper manufacturing procedures were employed according to ISO 5269-1:2005 [30]. Paper containing copper particles was produced by adding the particles during disintegration of the dry pulp (stage 1). The paper was prepared with cellulose pulp in a ratio of 1 DKL (Double Kraft Liner):1 OCC (Old Corrugated Container). After that, 0.6 g of each CuP was mixed per 1.21 g of pulp until 15 g of cellulose was obtained to manufacture 10 paper sheets for each presentation. The cellulose–copper mixture was homogenized in a disintegrator with 2 L of water at 30,000 rpm. Then, the sample was homogenized in 5 L of demineralized water for 5 min, and then the process was continued.

### 4.3. Characterization of Paper/CuPs

The optical properties of the color sheets were evaluated in an WR10QC Portable Colorimeter Fru^®^ (Shenzhen Chuangxin Instruments Co., Ltd., Shenzhen, China) to obtain the L*, a* and b* defined by CIELAB, where L* corresponds to lightness, a* represents a change in color from green (−) to red (+), and b* represents a change in color from blue (−) to yellow (+) [31]. The copper mass retained in the sheet was determined using Atomic Absorption Spectroscopy (AAS) (Agilent 240FS, Agilent Technologies, Santa Clara, CA, USA) from ANALAB. The percentage of retained copper was then calculated based on the mass of copper added during the disintegration stage. A Zeiss EVO MA10 Scanning Electron Microscope (SEM) (Carl Zeiss Microscopy GmbH, Jena, Germany) with a tungsten filament was used to observe the distribution of copper particles among the cellulose fibers. The samples were gold-coated using a Quorum Q150R ES Plus Sputtering System (Quorum Technologies Ltd., Laughton, East Sussex, UK) for 15 s at 22 mA.

X-ray fluorescence analysis (XRF) using a Thermo Scientific™ Niton™ XL3t (Thermo Fisher Scientific, Waltham, MA, USA) was employed to determine the elemental composition of the papers in the study of copper particle release after the “extreme washing” process. This process exacerbates the release conditions of metal particles adhered to cellulose fibers and quantifies the CuPs content released by calculating the difference in metal content in the paper before and after the process [25].

(1) Circular samples with a diameter of 2.5 cm were taken and measured using XRF; (2) the samples were immersed in 5 mL of water for 10 min, followed by vacuum filtration using a fritted glass funnel, then washed with three 5 mL aliquots of water; (3) the paper was dried in an oven at 50 °C for 30 min; (4) finally, the paper samples were reanalyzed using XRF.

### 4.4. Antimicrobial Analysis

The antibacterial activity of paper sheets containing copper particles was evaluated using two international standardized methods: ISO 20645:2004 and ISO 20743:2013.

Method 20645:2004 (Textile Fabrics—determination of antibacterial activity—agar diffusion plate test) is a standardized method that is used to evaluate the antibacterial activity of textile materials with diffusible antimicrobial agents. This test evaluates the antimicrobial performance by measuring the inhibition zone formed around the tested material. Briefly, a 2.54 cm circular sample was punched out from a 20 cm diameter paper sheet. Samples were handled with gloves immediately after fabrication and stored individually at room temperature, protected from light. Paper samples were placed on two-layer agar plates, with the lower layer containing only the agar medium while the upper layer was inoculated with the selected bacteria strain (10^6^ CFU/mL). Both sides of the paper were tested. After the incubation period (37 °C for 24 h), the Petri dishes were examined for signs of antimicrobial activity. Antibacterial effectiveness was determined by observing bacterial growth around the contact zone between the agar and the paper samples and measuring the inhibition zones. Paper without copper was used as a control. Antibacterial activity was calculated according to ISO 20645:2004 as detailed in Appendix A.

ISO 20743:2013 (Test Protocol—Determination of antibacterial activity of Textile Products) is a standardized quantitative method used to determine the efficacy of antibacterial textile products. The antibacterial activity is evaluated using the absorption method, in which the reduction in number of bacterial colonies on treated textiles is compared to that on untreated controls. In this case, 4 × 4 cm square samples were cut from a 20 cm diameter paper sheet. Samples were exposed to a target bacterial suspension (10^6^ CFU/mL) directly inoculated onto the surface of copper paper. To determine the number of viable colonies, both control and treated samples (3 each) were immediately washed with 20 mL of culture media after bacterial inoculation. The remaining 6 specimens (3 control and 3 testing samples) were placed in tubes and incubated at 37 °C ± 2 °C for 18 to 24 h with the test bacterial suspension. After incubation, the reaction was stopped by adding 20 mL of culture media to the tubes, followed by shaking to ensure proper mixing. Serial dilution and plating of the bacterial suspension from each tube were performed. The decrease in microbial growth was compared to that of the control specimens. The results are expressed as the average count of viable colonies that grew on the post-incubation plates. Appendix B details the calculations and determinations of the antibacterial activity effects.

## Figures and Tables

**Table 1:** Colorimetric parameters of the papers.

	L	a	b
Paper (Control)	61.9 ± 0.2	6.5 ±0.1	13.4 ± 0.1
Paper/CuNPs	59.8 ± 0.2	4.5 ± 0.1	13.7 ± 0.1
Paper/CuMPs	61.5 ± 0.2	6.5 ± 0.1	12.4 ± 0.1

**Table 2:** Antibacterial effect of paper sheets according to ISO 20645:2004 (average of three measurements).

Sample	Strain	Inhibition Zone [mm] Mean Value	Growth	Assessment
Paper	*S. aureus*	0.00 ± 0.00	Heavy	Insufficient effect
Paper/CuNPs	*S. aureus*	1.97 ± 0.29	None	Good effect
Paper/CuMPs	*S. aureus*	0.00 ± 0.00	Moderate	Insufficient effect
Paper	*E. coli*	0.00 ± 0.00	Heavy	Insufficient effect
Paper/CuNPs	*E. coli*	4.13 ± 0.29	None	Good effect
Paper/CuMPs	*E. coli*	0.00 ± 0.00	Heavy	Insufficient effect

**Table 3:** Antibacterial effect of paper sheets according to ISO 20743 (average of three measurements).

Sample	Strain	Incubation Time (h)	UFC/cm^2^	Log 10	A Value	Antimicrobial Activity	Reduction %
Paper	*S. aureus*	0	2.0 × 10^5^	5.3	-	-	-
		24	1.2 × 10^7^	7.1			
Paper/CuNPs	*S. aureus*	0	2.0 × 10^5^	5.3	6.1	Strong	100
		24	0	1			
Paper/CuMPs	*S. aureus*	0	2.0 × 10^5^	5.3	4.8	Strong	99.99
		24	2.0 × 10^2^	2.3			
Paper	*E. coli*	0	2.0 × 10^5^	5.3	-	-	-
		24	1.9 × 10^7^	7.3			
Paper/CuNPs	*E. coli*	0	2.0 × 10^5^	5.3	6.2	Strong	100
		24	0	1			
Paper/CuMPs	*E. coli*	0	2.0 × 10^5^	5.3	3.8	Strong	99.25
		24	3.5 × 10^3^	3.5			

## Data Availability

Data is contained within the article and Supplementary Materials.

## Supplementary Materials

The following supporting information can be downloaded at https://www.mdpi.com/article/10.3390/ijms26020480/s1.

## Appendix A

Antibacterial activity, according to ISO 20645, was calculated using the following formula:

H = (D − d)/2 (A1)

where H corresponds to the inhibition zone;

D: total sample diameter and inhibition zone;

d: sample diameter.

All of these measurements are expressed in mm.

The antibacterial effect is defined according to the criteria in Table A1 (ISO 20645).

**Table A1:** Antibacterial effect according to ISO 20645 criteria for antibacterial treatment.

Inhibition Zone [mm] Mean Value	Growth	Description	Assessment
>1	None	Inhibition zone exceeding 1 mm; no growth	Good effect
1–0	None	Inhibition zone up to 1 mm; no growth	
0	None	No inhibition zone; no growth	Limited efficacy
0	Slight	No inhibition zone; only some restricted colonies; growth nearly totally suppressed	
0	Moderate	No inhibition zone; compared to the control, growth reduced to half	Insufficient effect
0	Heavy	No inhibition zone; compared to the control, no growth reduction or only slightly reduced growth	

## Appendix B

Antibacterial activity, according to ISO 20743:2013, was calculated with the following formula:

(A2) $$A=\left(I_{g}C_{t}-I_{g}C_{o}\right)-\left(I_{g}T_{t}-I_{g}T_{o}\right)=F-G$$

where A corresponds to the value of the antibacterial activity;

F: count of viable bacteria in the control sample ($F=I_{g}C_{t}-I_{g}C_{O}$);

G: count of viable bacteria in the experimental samples ($G=I_{g}T_{t}-I_{g}C_{T}$);

IgTt: average common logarithm for the number of bacteria obtained from three experimental samples tested in triplicate after 18–24 h of incubation;

IgTo: average common logarithm for the number of bacteria obtained from three experimental samples tested in triplicate immediately after inoculation.

The value obtained from the formula above was compared with that obtained for controls using interpretation ranges (Table A2) as proposed by the Hohenstein Research Institute (2008).

**Table A2:** Interpretation ranges of antimicrobial activity proposed by the Hohenstein Research Institute.

Antimicrobial Activity	Specific Efficacy
None	<0.5
Slight	0.5 < 1
Significant	1 < 3
Strong	≥3
