# Supplementary material for: Copper-Modified Cellulose Paper: A Comparative Study of How Antimicrobial Activity Is Affected by Particle Size and Testing Standards
Source: Int J Mol Sci. 2025 Jan 8;26(2):480. doi: 10.3390/ijms26020480 (PMC11765064; doi:10.3390/ijms26020480)
Supplement: Supplementary file 1 [file ijms-26-00480-s001.zip › ijms-3396781-supplementary.pdf]

SUPPLEMENTARY MATERIALS

Copper-Modified Cellulose Paper: A Comparative Study of How Antimicrobial Activity Is Affected by Particle Size and Testing Standards

Sara Ramírez [1], Fabian Zuñiga [2], Alejandra Amenabar [1], Paulina Contreras [1], Viviana Benavides [1], Javiera Norambuena [1], Jessica Martínez [2,*] and Nataly Silva [1,*]

[1] Facultad de Diseño, Universidad del Desarrollo, Avenida Plaza 680, Las Condes 7610658, Santiago, Chile; sara.ramirez@usach.cl (S.R.); paulinacontreras@udd.cl (P.C.); aamenabar@udd.cl (A.A.); d.benavides01@ufromail.cl (V.B.); javiera.norambuena@upla.cl (J.N.)

[2] Centro de Medicina Regenerativa, Facultad de Medicina, Clínica Alemana-Universidad del Desarrollo, Avenida Plaza 680, Las Condes 7610658, Santiago, Chile; fabian0110@gmail.com

* Correspondence: jemartinez@udd.cl (J.M.); nrsilva@udd.cl (N.S.)

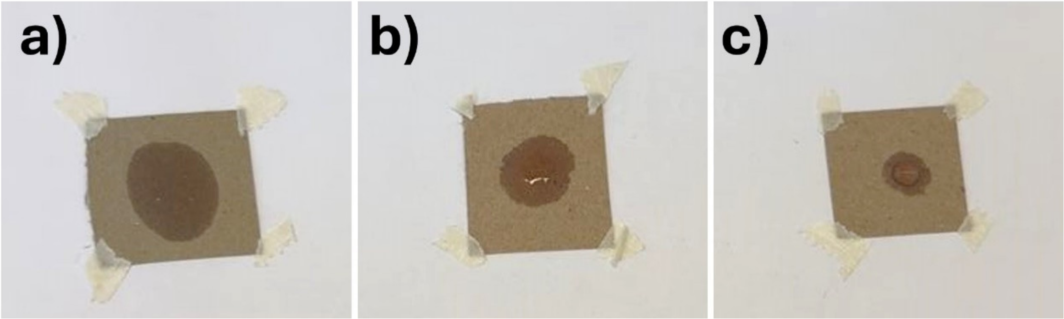

Figure S1. Photograph of water absorption 5 seconds after adding a 20 μL aliquot onto the paper. a) Paper/CuNP, b) Paper, and c) Paper/CuMP

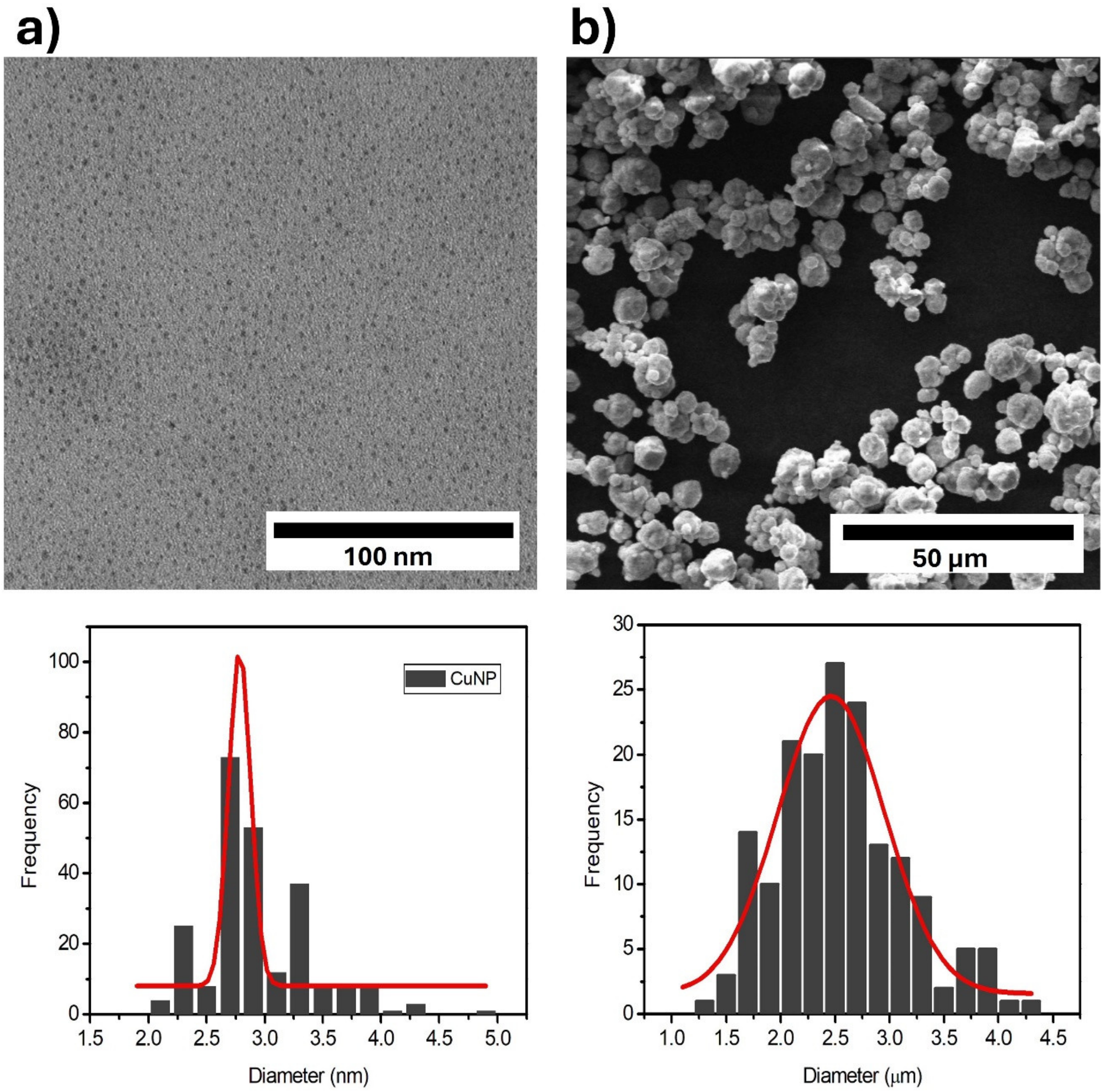

Figure S2. SEM micrographs and particle size distribution histograms in suspension of a) CuNP and b) CuMP.

Particle diameter in suspension: CuNP 2.7 ± 0.54 nm and CuMP 2.5 ± 0.8 µm
